# The CYP17 MSP AI (T-34C) and CYP19A1 (Trp39Arg) variants in polycystic ovary syndrome: A case-control study

**DOI:** 10.18502/ijrm.v17i3.4519

**Published:** 2019-05-29

**Authors:** Zohreh Rahimi, Ehsan Mohammadi M.Sc.

**Affiliations:** ^1^Medical Biology Research Center, Kermanshah University of Medical Sciences, Kermanshah, Iran.; ^2^Department of Clinical Biochemistry, Medical School, Kermanshah University of Medical Sciences, Kermanshah, Iran.

**Keywords:** *Cytochrome 17 (T-34C)*, * Cytochrome 19 (Trp39Arg)*, * Polycystic ovary syndrome*, * Sex steroid hormones*, * Sex hormone-binding globulin.*

## Abstract

**Background:**

Polycystic ovary syndrome (PCOS) is a common and chronic disorder of endocrine glands where genetic factors play a major role in the susceptibility to the disease. The cytochrome (CYP) 17 enzyme is essential for androgens biosynthesis. Also, the CYP19 enzyme converts the androgens to the aromatic estrogens.

**Objective:**

We aimed to investigate the association of CYP 17 MSP AI (T-34C) and CYP 19A1 (Trp39Arg) variants with the pathogenesis of PCOS in a population from Western Iran with Kurdish ethnic background.

**Materials and Methods:**

The present case-control study consisted of 50 patients with PCOS and 109 controls. The CYP17 T-34C and CYP19A1 (Trp39Arg) polymorphisms were identified by polymerase chain reaction-restriction fragment length polymorphism. The serum lipid and lipoprotein profile were detected by the Bionic Diagnostic Kits. Estradiol, dehydroepiandrosterone (DHEA), and sex hormone-binding globulin (SHBG) levels were measured using the chemiluminescent method.

**Results:**

The serum levels of estradiol and SHBG in PCOS patients were lower than controls (p < 0.001 and p = 0.06, respectively). However, the level of DHEA was higher (p = 0.01) in patients compared to controls. The higher frequency of CYP17 TC genotype in patients (30%) compared to controls (15.6%) was associated with 2.31-fold susceptibility to PCOS (p = 0.038). The frequency of CYP19 TC genotype was 6.4% in controls and 10% in patients (p = 0.42).

**Conclusion:**

The present study suggests that CYP17 TC genotype could be associated with the risk of PCOS. Also, the study indicated the sex steroid hormones level alteration and the lower level of SHBG in PCOS patients compared to healthy individuals.

## 1. Introduction 

Polycystic ovary syndrome (PCOS) is a common and chronic disorder of endocrine glands affecting up to 10% of the women at the age of fertility (1). The principal cause of this syndrome is not known, but studies have shown that genetic factors play a major role in susceptibility to the disease (2). The most important complications of the PCOS are increased level of androgens, infertility, hirsutism, dysfunction in ovulation and menstruation, and metabolic disorders (3, 4). The 17α-hydroxylase/17–20 lyase (CYP17A1) is a key enzyme in the pathway of androgen biosynthesis that catalyzes the conversion of pregnenolone to 17-hydroxy pregnenolone and the conversion of progesterone to 17-hydroxy progesterone in adrenal gland and ovary (5). The increased activity of this enzyme results in increased synthesis and secretion of androgens in PCOS (6). The CYP17A1 gene is located on chromosome 10q24.3 and has eight exons. There is a polymorphism at the promoter region of the CYP17 MSP AI (T-34C/ rs743572) that plays a role in the regulation of the gene expression. The presence of this polymorphism may lead to increased synthesis of androgens (6, 7). There are inconsistent reports about the role of CYP17 MSP Al (T-34C) polymorphism in susceptibility to developing PCOS (6, 8, 9). The CYP19A1 (aromatase) is a key enzyme in the pathway of estrogen biosynthesis from androgen, which is expressed in various tissues such as ovary, breast, fatty tissue, etc. (10, 11). The CYP19 gene is located on chromosome15q21.1 and contains 10 exons (12). A polymorphism in codon 39 of exon 2 of the CYP19 (Trp39Arg/rs2236722) leads to reduced activity of aromatase enzyme (13, 14). According to the literature, there is no available study to examine the role of CYP19A1 (Trp39Arg) polymorphism in the pathogenesis of PCOS.

In circulation, estradiol, testosterone, and dehydroepiandrosterone (DHEA) are transported through binding to the sex hormone-binding globulin (SHBG) and albumin that affect the bioavailable fraction of the hormones (15).

The aims of this study were to identify the association between CYP17 MSP AI (T-34C/rs743572) and CYP19 (Trp39Arg/rs2236722) polymorphisms with susceptibility to PCOS and also with lipid profile and sex hormone levels in a population from Western Iran with ethnic background of Kurds.

## 2. Materials and Methods

In this present case-control study, 50 women with confirmed PCOS (case) and 109 unrelated age-matched healthy individuals without PCOS (control) according to the Rotterdam criteria were enrolled (16). Patients were individuals who referred to the Kermanshah University of Medical Sciences clinic. The mean age of case group was 23.6 ± 5.3 yr (14–43 yr) and the controls were with the mean age of 22.3 ± 4.0 yr (18–33 yr, p = 0.11). Controls were selected from students and the staff of Kermanshah University of Medical Sciences that did not have a history of hyperandrogenism (the presence of hirsutism, acne or alopecia, and menstrual irregularity).

Two criteria of hyperandrogenism (the presence of hirsutism, acne or alopecia) and ovarian dysfunction (oligo- and/or an-ovulation and/or polycystic ovaries detected by ultrasound scans) were used for the diagnosis of PCOS. The exclusion criteria were diseases affecting androgens production such as congenital adrenal hyperplasia, androgen-secreting tumors, and also the intake of any medication that may affect the endocrine and biochemical parameters at least three months prior to enrolment.

Using the provided height and weight from each individual the body mass index was calculated. All women in this study were from the Kermanshah province of Western Iran with ethnic background of Kurds.

### Biochemical analysis

From each individual, a sample of venous blood (10 milliliters) was collected at 9 am of the day 5 of the menstrual cycle under standard conditions and used for biochemical and genetic analysis according to the standard protocol.

The levels of blood parameters such as fasting blood sugar (FBS), triglycerides, cholesterol, low-density lipoprotein-cholesterol (LDL-C), and high-density lipoprotein-cholesterol (HDL-C) were determined by using the Bionic Diagnostic Kits (Iran) on Mindray BS-480 Chemistry Analyzer (China). Serum estradiol level in the day 5 of the menstrual cycle (follicular phase), the DHEA, and SHBG levels were detected by the chemiluminescent method using the Abbott Architect i1000 (Abbott Laboratory, USA).

### Genotype analysis

The standard method of phenol-chloroform was used for extraction of DNA from the obtained whole blood of each individual (17).

For detection of the CYP17 (T-34C) genotypes the forward primer of 5'- CAT TCG CAC TCT GGA GTC -3' and the reverse primer of 5'- AGG CTC TTG GGG TAC TTG -3' were used. The PCR cycling conditions were the initial denaturation at 94${}^{\circ }$C for 5 min followed by 30 cycles of 94${}^{\circ }$C for 60 sec, 56${}^{\circ }$C for 60 sec, 72${}^{\circ }$C for 60 sec. At the end of cycles a final elongation step at 72${}^{\circ }$C for 7 min was applied. Five microliters of the resulting PCR products were electrophoresed on a 2% agarose gel. The 414-bp PCR product was digested with MSP AI restriction enzyme. The presence of C allele resulted in digestion of the 414-bp fragment to two fragments of 290- and 124-bp. While no digestion was occurred in the presence of T allele and the 414-bp PCR product remained intact (18).

The CYP19A1 (Trp39Arg) polymorphism was identified using the two-pair primers of the forward primer 1: 5'-ATC TGT ACT GTA CAG CAC C-3', and the reverse primer 1: 5' **-**ATG TGC CCT CAT AAT TCC G-3', the forward primer 2: 5'-GGC CTT TTT CTC TTG GTG T-3' and the reverse primer 2: 5'-CTC CAA GTC CTC ATT TGC T-3'. PCR reaction was carried out with a total volume of 25 microliters containing 100 ng of genomic DNA, 2.5 μl of 10X PCR buffer, 200 μM dNTPs, 1.5 mM MgCl${}_{2}$, 1 unit of Taq DNA polymerase, and 20 pmol of each primer. The PCR cycling conditions, after the initial denaturation at 94${}^{\circ }$C for 5 min, were as follows: 94${}^{\circ }$C for 60 sec, 56${}^{\circ }$C for 60 sec, 72${}^{\circ }$C for 60 sec (30 cycles), followed by the final elongation at 72${}^{\circ }$C for 7 min. The resulting PCR products were visualized using electrophoresis on a 2% agarose gel. In the presence of T allele, a fragment with 200-bp is produced and the presence of C allele produces a 264-bp fragment. Also, a common 427-bp fragment was observed in the PCR products of both alleles (19).

### Ethical consideration

All individuals agreed to participate in the study and signed a written informed consent before participation. The Ethics Committee of Kermanshah University of Medical Sciences approved the study (code: 96500). The study was in accordance with the principles of the Declaration of Helsinki II.

### Statistical analysis

The frequencies of alleles were calculated by the chromosome counting method. The frequencies of CYP17 T-34C and CYP19A1 (Trp39Arg) genotypes and alleles have been compared between patients and controls using chi-square test. Odds ratios (OR) as an estimation of relative risk of the disease and 95% confidence intervals (CIs) were obtained by SPSS logistic regression. The interaction between alleles of CYP17 and CYP19 was determined using logistic regression model. The independent-sample *t*-test was used to find the correlation of biochemical parameters with the studied polymorphisms between groups. Two-tailed Student's *t*-test and ANOVA analysis were also used for comparison of quantitative data. Statistical significance was assumed at the p-value < 0.05. Statistical analysis was performed using the statistical package for social sciences (SPSS) logistic regression (SPSS, Inc., Chicago, IL) version 16.0.

## 3. Results

The clinical and biochemical characteristics of patients and controls are demonstrated in Table I. Comparing biochemical parameters between patients and controls indicated a significantly lower level of estradiol in patients compared to controls (p < 0.001). Also, a near to significant lower level of SHBG was observed in patients than controls (p = 0.06). However, the DHEA level was significantly higher in patients compared to controls (p = 0.01) (Table I).

The distribution of CYP17 T-34C genotypes was in Hardy–Weinberg equilibrium in both patients ($\chi^{2}$ = 1.99, p > 0.1) and controls ($\chi^{2}$ = 0.78, p > 0.1). Also, the distribution of genotypes of CYP19 T > C was in Hardy–Weinberg equilibrium in both PCOS patients ($\chi^{2}$ = 0.12, p > 0.1) and controls ($\chi^{2}$ = 0.14, p > 0.1). Table II demonstrates the distribution of CYP17 MSP AI (rs743572) and CYP19 (rs2236722) genotypes and alleles in PCOS patients and controls. The frequency of CYP17 TC genotype was 30% in patients compared to 15.6% in controls that was significantly associated with 2.31-fold susceptibility to PCOS (OR = 2.31 (95% CI 1.04–5.14, p = 0.038). The genotype of CYP17 CC was not detected among both patients and controls. There was a significantly higher frequency of CYP17 C allele in patients (30%) compared to controls (15.6%, p = 0.035) (Table II). The frequency of CYP19 TC genotype was 6.4% in controls and 10% in patients (p = 0.42). Although, the presence of CYP19 TC genotype increased the risk of PCOS but it did not reach to a statistical significance [OR = 1.61 (95%CI: 0.48–5.37, p = 0.43)] (Table II). Similar to CYP17 polymorphism, no CYP19 CC genotype was detected in both patients and controls. In Table III, the biochemical parameters have been compared between various genotypes of CYP17 MSP AI in patients and controls separately. Among the controls, there was a significantly higher level of FBS in the presence of CYP17 TC genotype (86.1 ± 39.1 g/dl) compared to TT genotype (74 ± 10.2 g/dl, p = 0.01) (Table III). We observed lower levels of estradiol and SHBG and a higher concentration of DHEA in PCOS women carrying the CYP17 TC genotype compared to those carrier of TT genotype that did not reach to a statistical significant. Comparing the biochemical and hormonal parameters between the patients and controls carriers of CYP17 TT or TC genotype indicated the absence of a significant difference except for significantly higher estradiol level in controls with TT genotype than patients with this genotype (133.7 ± 91.6 vs. 70.9 ± 44.2 pg/ml (p = 0.01). Table IV indicates haplotype analysis of two CYP17 MSP AI and CYP19 T > C polymorphisms in patients and controls. Haplotype analysis demonstrated that in patients compared to controls, there was a significantly higher percentage of concomitant presence of CYP17T and CYP19C alleles than the presence of CYP17C and CYP19T as reference haplotype ($\chi^{2}$ = 5.09, p = 0.024) (Table IV).

**Table 1:** The clinical and biochemical characteristics of patients with PCOS and controls.


**Variables**	**Patients (n = 50)**	**Controls (n = 109)**	**p**
Age (years)	23.6 ± 5.3	22.3 ± 4	0.11
BMI (Kg/m${}^{2}$)	23.7 ± 4.9	22.3 ± 3.7	0.4
FBS (mg/dl)	78.5 ± 13.2	75.9 ± 14.8	0.36
Cholesterol (mg/dl)	131.1 ± 32.7	124.1 ± 28.6	0.17
TG (mg/dl)	87.9 ± 51.5	76.9 ± 33.8	0.11
HDL-C (mg/dl)	45.5 ± 11.7	41.6 ± 10.1	0.03
LDL-C (mg/dl)	73.9 ± 26.5	70.5 ± 21	0.38
Estradiol (pg/ml)	70 ± 45.5	130 ± 92	< 0.001
SHBG (pg/ml)	52.2 ± 24.5	64.6 ± 42.9	0.06
DHEA (pg/ml)	278.7 ± 148.7	215.4 ± 142	0.01
Systolic blood pressure (mmHg)	100.2 ± 11.6	99.6 ± 13.3	0.79
Diastolic blood pressure (mmHg)	71.6 ± 9.9	73 ± 10.5	0.42
The student's t-test was used for comparing parameters between two groups. Data presented as mean ± SD			
Note: BMI: Body mass index; FBS: Fasting blood sugar; TG: Triglyceride; HDL-C: High-density lipoprotein cholesterol; LDL-C: Low-density lipoprotein cholesterol; SHBG: Sex hormone-binding globulin; and DHEA: Dehydroepiandrosterone			

**Table 2:** The frequency of CYP19 T > C (rs2236722) and CYP17 MSP AI (rs743572) genotypes and alleles in patients with PCOS and controls.


**Genotypes**	**Controls** **(n = 109)**	**Patients** **(n = 50)**	**$\chi^{2}$**	**p**
CYP19T > C				
TT	102 (93.6)	45 (90)	
TC	7 (6.4)	5 (10)	<brow>-2</erow>0.62	<brow>-2</erow>0.42
OR = 1.61 (95% CI: 0.48–5.37, p = 0.43)				
Alleles				
T	204 (93.6)	90 (90)	
C	14 (6.4)	10 (10)	<brow>-2</erow>0.62	<brow>-2</erow>0.42
CYP17 MSP AI				
TT	92 (84.4)	35(70)	
TC	17 (15.6)	15 (30)	<brow>-2</erow>4.42	<brow>-2</erow>0.035
OR = 2.31 (95% CI: 1.04–5.14, p = 0.038)				
Alleles				
T	184 (84.4)	70 (70)	
C	34 (15.6)	30 (30)	<brow>-2</erow>4.42	<brow>-2</erow>0.035
OR = 2.31 (95% CI: 1.04–5.14, p = 0.038)				
Data presented as n (%).				

**Table 3:** Comparison of biochemical parameters according to CYP17AI genotypes in all studied individuals


**Parameters**	**Patients (n = 50)**			**Controls (n = 109)**		
	**TC**	**TT**	**p**	**TC**	**TT**	**p**
BMI (Kg/m${}^{2}$)	24.7 ± 5.6	23.3 ± 4.6	0.34	20.9 ± 4.4	22.5 ± 3.5	0.10
FBS (mg/dl)	76.6 ± 11.6	79.4 ± 13.9	0.49	86.1 ± 39.1	74 ± 10.2	0.01
TG (mg/dl)	77.8 ± 43.7	92.4 ± 54.6	0.36	68.6 ± 22.1	78.5 ± 35.4	0.28
HDL-C (mg/dl)	47.6 ± 13.9	44.6 ± 10.6	0.42	43.2 ± 10.9	41.3 ± 10	0.49
LDL-C (mg/dl)	75.9 ± 31.5	73 ± 24.5	0.73	69.4 ± 14.6	70.7 ± 22.1	0.82
Cholesterol (mg/dl)	129.8 ± 36.9	131.7 ± 31.3	0.85	124.4 ± 19.8	124 ± 30	0.96
Estradiol (pg/ml)	67.7 ± 50.1	70.9 ± 44.2	0.83	108.8 ± 95	133.7 ± 91.6	0.33
SHBG (pg/ml)	49.9 ± 20.9	53.2 ± 26.2	0.66	66.5 ± 30.3	64.3 ± 45	0.85
DHEA (pg/ml)	303.3 ± 151.5	267.9 ± 148.4	0.44	200.6 ± 148.9	218.1 ± 141.4	0.6
Systolic blood pressure (mmHg)	102.6 ± 10.3	99.1 ± 12.2	0.33	105.2 ± 13.2	98.5 ± 13.1	0.056
Diastolic blood pressure (mmHg)	72.6 ± 9.6	71.1 ± 10.2	0.62	74.1 ± 8.7	72.8 ± 10.9	0.64
Data presented as mean ± SD.						
Note: BMI: Body mass index; FBS: Fasting blood sugar; TG: Triglyceride; HDL-C: *High-density lipoprotein cholesterol;* LDL-C: *Low-density lipoprotein cholesterol; SHBG: *Sex hormone-binding globulin; and DHEA: Dehydroepiandrosterone						

**Table 4:** The synergism effect of CYP17 MSP AI (rs743572) and CYP19A1 (rs2236722) polymorphisms in patients with PCOS and controls


**CYP17AI **	**CYP19A1**	**Controls n = 109**	**Patients n = 50**	**$\chi^{2}$**	**p**
T	T	17 (15.6%)	13 (26%)	–	–
C	T	85 (78%)	32 (64%)	*2.87 **5.09	*0.09 **0.024
C	C	7 (6.4%)	3 (6%)	*0.55	0.45
T	C	0	2 (4%)	*2.41	0.12
Note: Overall: $\chi^{2}$ = 7.24; p = 0.064; *Compared to reference; and **Compared to CYP17T and CYP19C					

## 4. Discussion

A genetic basis for susceptibility to PCOS has been suggested (2). The enzyme of CYP17 is essential for androgen biosynthesis in the adrenal gland and ovary. The presence of CYP17 C allele (the A2 allele, rs743572) has been hypothesized to create an additional Sp1-binding site which increased the activity of promoter and enhanced the gene expression (20).

The current study revealed that the CYP17 TC genotype was associated with 2.31-fold increased risk of PCOS. It seems increased risk of PCOS in the presence of CYP17 TC genotype could be attributed to higher promoter activity in the presence of this genotype compared to TT genotype. The CYP17 CC genotype was not detected among our studied samples because the homozygosity for the C allele is rare in the populations (21, 22).

A study carried out by Pusalkar and colleagues (23) demonstrated the high frequency of CYP17 C allele in PCOS patients compared to controls. Similar to the report of Pusalkar and colleagues (23), the current study indicated that the frequency of C allele was significantly increased in women with PCOS compared to the controls and the CYP17 (T-34C) polymorphism enhanced the risk of PCOS.

However, in a study conducted in China, the frequency of genotypes of the CYP17 (T-34C) polymorphism was not significantly different between women with PCOS and healthy controls (5). A meta-analysis by Li and coworkers (6) demonstrated the absence of relationship between CYP17 (T-34C) variants and increased risk of PCOS among Caucasian and Asian populations. Also, some studies have shown no correlation between CYP17 (T-34C) alleles and PCOS (8, 24, 25). Controversial reports related to the association of CYP17 (T-34C) gene polymorphism with susceptibility to PCOS might be due to various frequencies of the polymorphism among different populations and the effect of sample size.

In our study non-significant lower levels of estradiol and SHBG and a higher concentration of DHEA in PCOS women with CYP17 TC genotype compared to women with TT genotype was detected. Although, the presence of CYP17 (T-34C) polymorphism enhances the promoter activity and increases the gene expression with subsequent elevation of androgen production, it has not been confirmed by experimental studies (26, 27). The mechanism by which the CYP17 (-T34C) polymorphism might increase the serum level of sex steroid hormones has not been identified. However, it might be due to the effects that have not yet been elucidated or the polymorphism could be in linkage disequilibrium with another polymorphism that leads to the CYP17 enzyme function alteration (27).

In the present study, no significant difference was found in the frequency of different CYP19A1 (Trp39Arg) genotypes between patients with PCOS and controls. Also, the association of CYP19 TC genotype with the risk of PCOS did not reach to a statistical significance. There is no available study to examine the role of CYP19 A1 (Trp39Arg) variants in the pathogenesis of PCOS. In the present study, the CYP19 A1 (Trp39Arg) was not associated with the risk of PCOS. However, a different polymorphism in CYP19 gene (rs2414096) has been reported to be associated with the risk of PCOS among Iranian women (28).

Haplotype analysis indicated the lack of significant difference between CYP17C/CYP19C haplotype frequencies compared to the reference haplotype of CYP17T/CYP19T. However, concomitant presence of CYP17T and CYP19C alleles had significantly higher frequency than the combined presence of CYP17C and CYP19T as the reference haplotype in patients.

There is high prevalence of lipid abnormalities in women with PCOS (29). However, in our study, we did not detect a significant difference in lipid profile between PCOS patients and controls.

## 5. Conclusion

In conclusion, we found that the CYP17 (T-34C) polymorphism increased the risk of PCOS. However, our study indicated that the CYP19A1 (Trp39Arg) polymorphism might not be a risk factor for susceptibility to PCOS in our population. Also, the present study detected an association between PCOS with lower serum levels of SHBG and estradiol and higher DHEA concentration.

##  Limitation

The limitation of the present study is the low sample size especially in patients with PCOS.

##  Future Perspective

During the next 5–10 yr, more studies will be conducted to elucidate the role of CYP17 (T-34C) and CYP19A1 (Trp39Arg) gene variants and their influence on the activities of CYP17 and CYP19 enzymes in PCOS among various populations. Also, the molecular mechanism of CYP17 (T-34C) and CYP19A1 (Trp39Arg) gene expression in relation to modulatory effects of epigenetic and environmental factors will be more understood.

##  Conflict of Interest

There authors declare no conflict of interest.
